# Utilization of Polymer Concrete Composites for a Circular Economy: A Comparative Review for Assessment of Recycling and Waste Utilization

**DOI:** 10.3390/polym13132135

**Published:** 2021-06-29

**Authors:** Hatem Alhazmi, Syyed Adnan Raheel Shah, Muhammad Kashif Anwar, Ali Raza, Muhammad Kaleem Ullah, Fahad Iqbal

**Affiliations:** 1 National Center for Environmental Technology (NCET), Life Science and Environment Research Institute (LSERI), King Abdulaziz City for Science and Technology (KACST), Riyadh 11442, Saudi Arabia; halhazmi@kacst.edu.sa; 2 Department of Civil Engineering, Pakistan Institute of Engineering and Technology, Multan 66000, Pakistan; kashifanwar723@gmail.com; 3 Department of Civil Engineering, University of Engineering and Technology, Taxila 47080, Pakistan; araza4846@gmail.com; 4 Department of Civil Engineering, University of Lahore, Lahore 54000, Pakistan; muhammad.kaleem1@ce.uol.edu.pk; 5 Department of Mechanical and Structural Engineering and Materials Science, University of Stavanger, NO-4036 Stavanger, Norway; fahadmeyo@gmail.com

**Keywords:** circular economy, recycling, green concrete, polymer concrete composites, sustainability, industrial wastes, pozzolanic binders, mechanical properties, durability

## Abstract

Polymer composites have been identified as the most innovative and selective materials known in the 21st century. Presently, polymer concrete composites (PCC) made from industrial or agricultural waste are becoming more popular as the demand for high-strength concrete for various applications is increasing. Polymer concrete composites not only provide high strength properties but also provide specific characteristics, such as high durability, decreased drying shrinkage, reduced permeability, and chemical or heat resistance. This paper provides a detailed review of the utilization of polymer composites in the construction industry based on the circular economy model. This paper provides an updated and detailed report on the effects of polymer composites in concrete as supplementary cementitious materials and a comprehensive analysis of the existing literature on their utilization and the production of polymer composites. A detailed review of a variety of polymers, their qualities, performance, and classification, and various polymer composite production methods is given to select the best polymer composite materials for specific applications. PCCs have become a promising alternative for the reuse of waste materials due to their exceptional performance. Based on the findings of the studies evaluated, it can be concluded that more research is needed to provide a foundation for a regulatory structure for the acceptance of polymer composites.

## 1. Introduction

**Figure 1:**
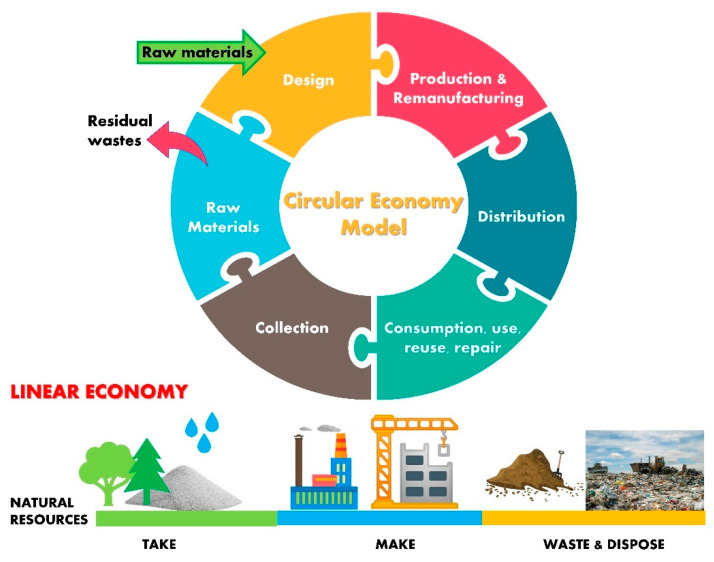
Circular economy cycle and linear economy model.

The growing climate challenges and the scarcity of new natural resources in construction projects have moved the research momentum towards sustainability. The concept of a ‘circular economy’ (CE) has become one of the effective ways of achieving long-term sustainability [1,2,3]. The current conventional economy model is centered on the approach of the manufacture, use, and then disposal of materials (Figure 1). CE is defined as the paradigm that has the potential to transform, protect, and generate revenues, resources, and materials back into circulation in the best possible way to be ecofriendly and cost effective. The concept of a circular economy is presented in Figure 1 for a better understanding of the circular model steps and the conventional model. In a linear model, we use resources to make a product and after use it will be thrown into the environment, causing environmental pollution. The CE seeks to build a closed-loop process and decrease resource use by promoting the persistent use of resources by recycling and reutilization instead of dumping [4,5,6]. Solid waste management raises a tricky challenge around the globe, especially to developed and emerging nations, caused by industrial progress, construction surges, urban growth, and the living environment [7,8]. The increase in legislation to mitigate the carbon footprint, reduce carbon emissions, and restrict landfill areas has boosted the interest of researchers towards geopolymer concrete in the construction sector. The utilization of waste materials has been endorsed in order to fulfill the construction requirements by reducing the consumption of natural resources [9,10,11,12]. Moreover, construction industries are encouraging sustainable and green construction as specified by the Leadership in Energy and Environmental Design (LEED).

Typical concrete has some drawbacks, such as utilization of natural resources in an excessive quantity, low compressive properties at an early age, and environmental hazards [13,14,15]. Geopolymer concrete has several advantages, such as improved concrete strength properties, a low carbon footprint, and reduced consumption of natural resources to name but a few [16]. The poor quality of locally available materials, systemic lock-in, higher construction costs, and technical problems are the main barriers against polymer concrete usage in the construction industry [17,18,19]. In addition, there is a need to introduce and promote the utilization of new and smart materials in order to protect natural materials and reduce the carbon footprint for the production of green and sustainable concrete [20]. Polymer concrete composites are an innovative approach to promoting and mitigating environmental aspects in the context of the extraction of new materials, design mix proportions, and design codes for the construction and rehabilitation of concrete structures [21,22,23].

Polymer concrete is a construction material made from a monomer/aggregate product that has been polymerized. The aggregates are bound together by the polymer matrix monomer, and the produced composite is known as concrete. The idea of polymer concrete started in the late 1950s. These materials were designed to be used as a substitute for cement in specific construction applications. Polymer concrete was first used for construction cladding and other applications. It was widely adopted as a repair material afterward due to its fast cure time, superior bonding properties, ability to reinforce steel, improved strength, and toughness [24,25]. Polymer concrete, similarly, to traditional concrete, can be strengthened with various types of fibers to improve its mechanical characteristics. Steel, glass, polypropylene, and nylon fibers have also been used in the past. A significant number of papers have been published on the impact of adding different forms of fibers into polymer concrete to improve it. The different types of fibers (steel fibers, glass fibers, carbon fibers, and polyester fibers) were used in various proportions to improve the properties of polymer concrete [26,27,28]. The incorporation of glass fibers into polymer concrete is used to enhance the post-peak behavior [29]. A few studies have shown that silane processing of glass fibers prior to application in polymer concrete improves the mechanical properties by up to 25% [30]. In order to minimize the drying shrinkage in the aggregate blend and thus improve the properties of polymer concrete, a micro filler is often applied to a mix. Calcium carbonate, fly ash, and silica fume have also been used in the past. Fly ash is a by-product of coal combustion in power plants that is employed as a filler, and it is widely available and has been shown to improve mechanical properties and minimize water absorption when used in polymer concrete [31]. Chemical resistance is one of the key advantages of polymer concrete composites over ordinary concrete. During the hydration process, Portland cement develops voids and cracks in ordinary concrete. Open gaps in polymer concrete can be eliminated by using a variety of resins, such as polyester-styrene, epoxy, and vinyl ester. Polymers are also hydrophobic by nature and can withstand harsh conditions [32,33]. On the other hand, PCCs are commonly flammable and degrade the structural as well as bonding properties at high temperatures [34,35]. However, due to a lack of experimental evidence of the fire resistance and suitability of PCCs, it is extremely difficult to choose the most suitable patch repair ingredients when repairs are desired [36,37]. As a result, their performance at high temperatures when exposed to fire must be clarified. However, there are limited data available in this area [38,39]. To satisfy the fire resistance criteria, the behavior of such materials at high temperatures must be studied based on a detailed analysis of their mechanical characteristics [38].

**Figure 2:**
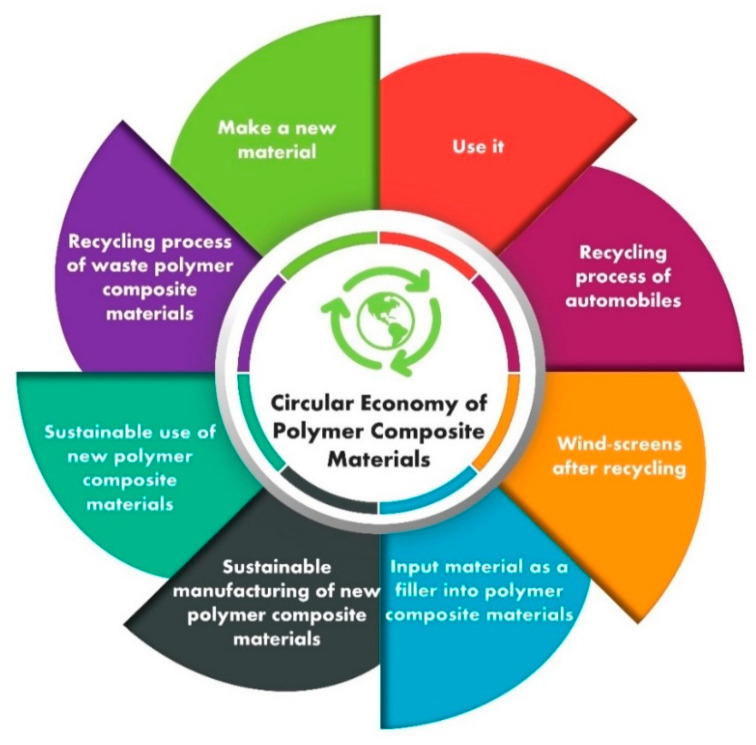
Circular economy concept for a polymer composite material.

Waste materials from steel industries are also rich in calcium and silica resources, which are important for the production of various calcium silicates and also mineralogical forms of calcium silicate. Rice husk and bagasse are examples of biomass waste that can be turned into ashes, which contain around 80–90% amorphous silica. For polymer concrete applications, this amorphous type of silica can save money. It is a known fact that waste materials such as fly ash from power stations have excellent pozzolanic characteristics and are being replaced by 30% in cement manufacturing. Moreover, fly ash in amounts up to 100% is being used as a building material owing to a process called geo-polymerization. The synergistic strategy to reuse industrial waste will serve as a model for developing building materials that reduce CO_2_ emissions. The accessibility of raw materials, the performance of the system, and the latest synthesis results all support the use of the polymer concrete model for the development of sustainable infrastructure materials. Reports have been published on a variety of curing methods, such as ambient temperature curing, elevated temperature curing, and water curing. The earlier studies have shown that after one day of soaking at room temperature, polymer concrete reaches about 70% to 75% of its strength. Normal concrete normally produces a 20% strength gain after one day of curing [29,40]. In precast uses, an initial strength gain is necessary as it allows for the structural elements to more quickly withstand higher stresses caused by form-stripping, carrying, delivery, and installation activities. After a seven-day period of dry curing, the strength of the concrete in polymer concrete promises to be fairly constant [41]. Composites incorporating wastes or by-products, such as FA, perlite dust, waste glass, and aggregate residual mineral dust, were put through a series of tests [42,43,44,45,46]. The effect of such composites, especially waste glass, in terms of mechanical properties and durability at different ages still remains unclear. Hence, there is a need to explore the mechanical behavior, durability performance, and economic analysis of sustainable polymer concrete composites made from different waste materials to close the research gaps. Hence, our objective is to highlight sustainable products, smart developments, and research on new ecofriendly materials through the circular economy concept as presented in Figure 2.

This review study aims to:

- Review the potential advantages of polymer concrete composites for the climate, then present a summary of the most important results concerning their mechanical properties, such as compressive, flexural, and splitting tensile strength.
- Examine the durability performance of polymer-modified concrete under a variety of adverse climatic conditions, and study its structural properties based on research findings.
- Collect data from earlier studies that used SCMs and industrial byproducts to partially replace conventional concrete for a better comparative analysis of their mechanical properties and then propose rankings that will help with the selection of SCM products for sustainable concrete production.

## 2. Common Wastes for Polymer Concrete

Agricultural, municipal, and industrial wastes are used as supplementary cementitious materials in making polymer concrete. The physical and chemical properties of the used waste materials were comprehensively studied and analyzed in terms of their pozzolanic properties for potential use in sustainable concrete [47,48].

### 2.1. Use of IW-I in Polymer Concrete

Many researchers have investigated the incorporation of the industrial waste (IW)-I product rice husk ash as a cement substitution or as a fine aggregate (sand) in concrete structure projects [47,49,50,51,52,53,54]. Rice husk ash (RHA) provides several tangible profits. Some of the advantages include improving the microstructure, reforming the void structure, and increasing the strength at the initial stage by decreasing the width of the ITZ in both aggregates and paste [55,56,57]. To improve the pozzolanic actions in concrete, several studies suggest the utilization of the optimum percentage for RHA mainly due to the utilization of different components in conjunction with RHA, variability in the manufacturing method, and applications. The connection between the particle diameter and the pozzolanic behavior of RHA has not been fully investigated. Despite this, several research studies are available on the utilization of RHA as a pozzolanic material in mortar and concrete. Previous studies have endeavored to clarify their association with varying degrees of performance. A positive relation was observed between the Blaine specific surface area (SSA) of RHA and cementitious characteristics; however, a negative correlation was seen in the case of a median particle size (d_50_) [58]. Conversely, the multi-layered, angular, and amorphous surface of RHA has been established as the predominant factor that controls the chemical reaction [59].

**Figure 3:**
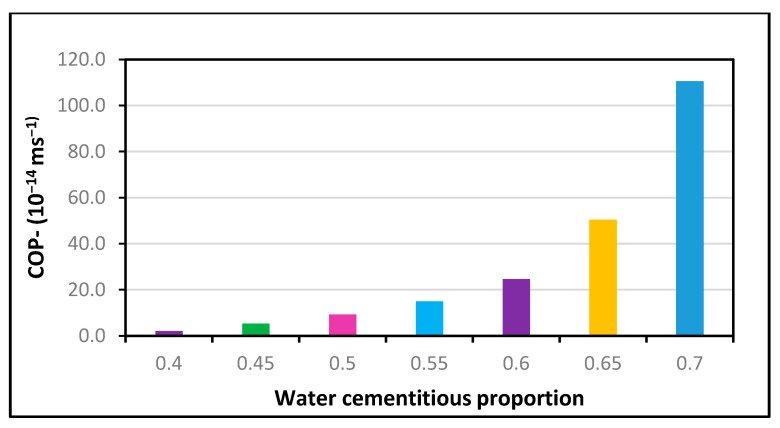
Relationship between the coefficient of permeability and the w/c proportion.

Detailed studies have shown that the size of the particle, its specific surface area, the W/C ratio, and the cement substitution level influence the cementitious properties of RHA. Similarly, it was reported that the impact of the specific surface area of RHA quite often overwhelms that of the particle size [59,60,61]. Givi et al. [60] found that the maximum compressive strength was obtained with a RHA particle size of 5 μm and a specific surface area of 36.47 m^2^/g in comparison with a RHA particle size of 95 μm and a specific surface area of 24 m^2^/g. Another research study reported the highest compressive strength of 51.8 MPa with a RHA particle size of 11.5 μm and a large surface area of 30.4 in a similar manner [59]. Compared with control samples, higher values (CS) were obtained with the relevant sizes of particles (31.3 and 18.3 μm), BET specific surface areas of 27.4 and 29.1, and a respective $CS_{28}$ of 48.4 and 50.2 MPa. These findings of the aforementioned studies provide evidence in terms of the superior effect of RHA’s specific surface area upon the pozzolanic actions and concrete strength. Likewise, low-permeability concrete is produced while using a design mix having a decreased water ratio or an increased cement ratio. High-strength concrete is less pervious than low-strength concrete. The concrete structure will be more durable as a result of this. The correlation between the ratio of water and the coefficient of permeability in concrete is shown in Figure 3. However, when the water–cement proportion is increased, the overall compressive strength of concrete will drop. An excessive amount of water results in a dilute paste with higher porosity at the micro scale. This will deteriorate the concrete, causing cracks or shrinkage problems. The surplus water in the concrete is absorbed by the coarse aggregates and cement particles. If there is an excessive amount of concrete mix, this consumption becomes difficult to control. As a result, multiple water channels are formed, causing the surface to bleed. This contributes to poor zones in concrete that are prone to failure when placed under a loading condition. A decreased water proportion can help to produce concrete with superior strength and quality. However, the water content alone is insufficient to produce better concrete. A proper mix percentage, good quality aggregates, and binding ingredients help to make a perfect combination.

**Figure 4:**
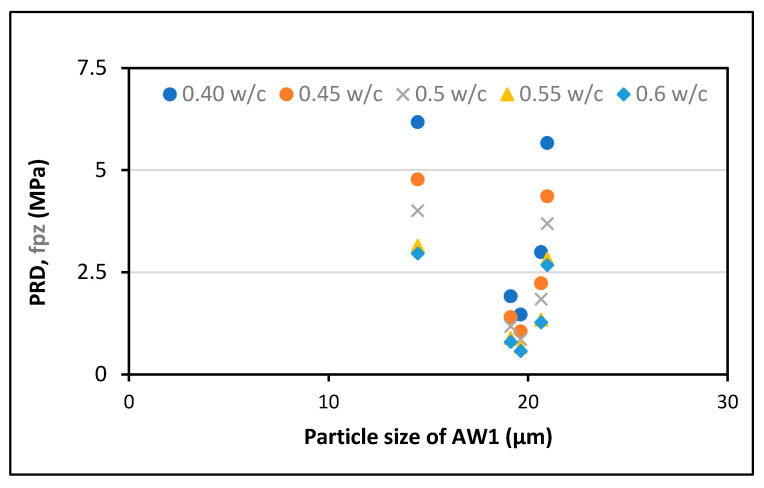
Discrepancy trends of the pozzolanic impact with the particle diameter and w/c ratio.

**Figure 5:**
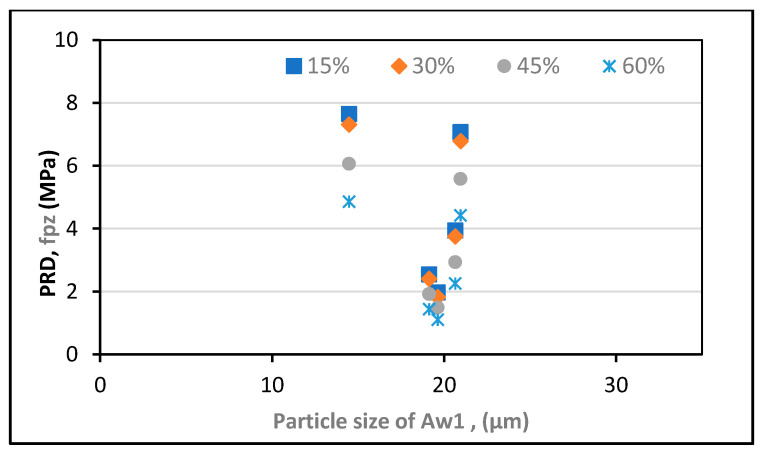
Discrepancy trends of the pozzolanic part with the particle diameter at varying percentages.

**Figure 6:**
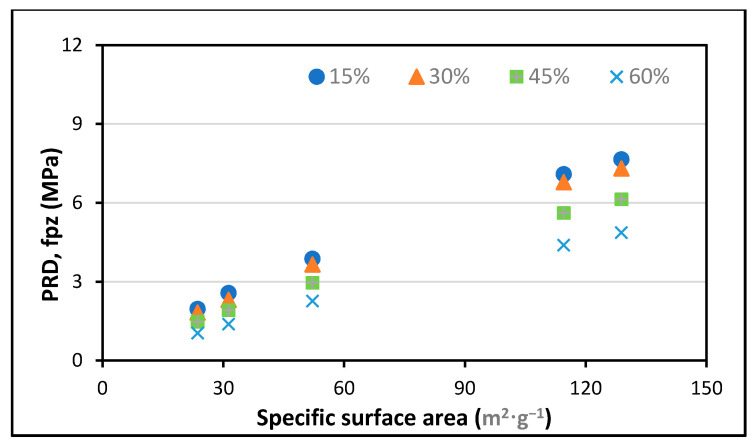
Discrepancy trends of pozzolanic reactions of RHA with BET SSA and the percentage of additives.

**Figure 7:**
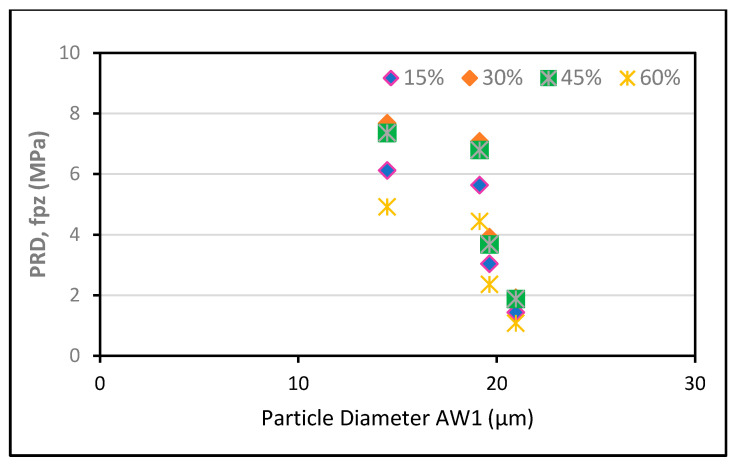
Discrepancy trends of pozzolanic involvement of RHA with different percentages at varying particle diameters.

**Figure 8:**
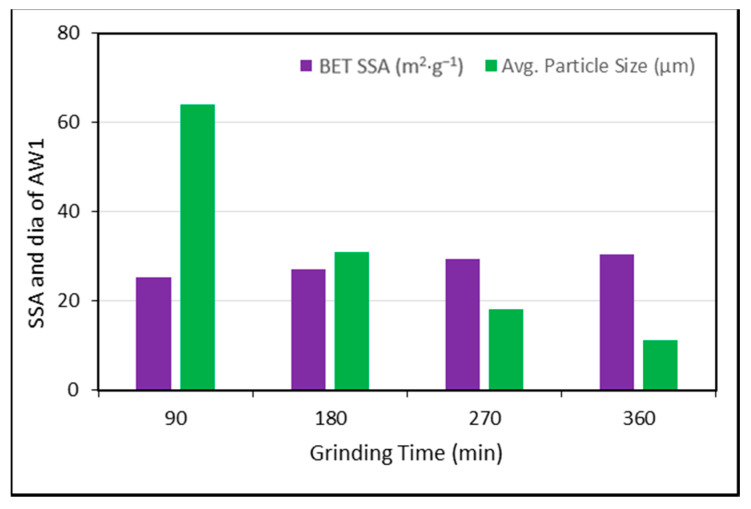
Evaluation of the pozzolanic involvement of RHA in SSA and particle size with grinding time.

For analysis, the experimental results provided by Zunino and Lopez [61] were used. RHA was purchased from various vendors with varying amorphous contents of d_50_, BET SSA, and SiO_2_. The experiment was performed at a cement replacement rate of 20% RHA and a w/c ratio of 0.5, and higher modification ratios were applied using the abovementioned formulas. The findings show that RHA’s pozzolanic contribution changes with the average particle size, the grinding time, the w/c ratio, the SSA, and the cement substitution dosages of RHA, as displayed in Figure 4, Figure 5, Figure 6, Figure 7 and Figure 8.

Furthermore, the optimum pozzolanic output was observed at a 30% substitution level of cement with an average particle size of 14.467 μm and a w/c ratio of 0.35. This result was consistent with the highest results found in another study [62]. Additionally, it was noticed that the pozzolanic performance enhanced as the BET SSA grew and the average particle size reduced. To ensure the optimum pozzolanic involvement of RHA in concrete, RHA particles having a smaller surface area and diameter are used. This means that if the RHA has a larger SSA, particles of smaller sizes would have the opportunity to provide more pozzolanic participation in concrete. Moreover, as most of the reported literature studies were conducted under sub-optimal test conditions, the capacity of RHA in concrete is still not completely understood. Hence, more research is required to explore efficient and economical ways to increase the specific surface area of locally generated RHA to support its use and acceptance around the world.

For all median-size particles, researchers [61] measured an above-average effective surface value in comparison with the specific surface area Eff model proposed by this study [63]. Even though the two methods displayed a similar pattern at a particle size of 20.644 m, the findings were said to be different. A simpler method could use the $SiO_{2-Eff}$ to assess the RHA’s pozzolanic contribution to the concrete. This is also supported by the fact that $SiO_{2}$ is around 80% to 90% of the mass of RHA and the principal cause of pozzolanicity actions [60,62,64,65].

Moreover, close attention should be paid to the production line and molecular structure together with the other concrete/cement additives used in RHAC to increase RHA’s pozzolanic efficiency in concrete [57,66]. The overall grinding period of RHA is determined by the burning temperature, the flaming time, the type of combustion device involved, the pre-treatment process of RHA, the number of revolutions, and the grinder type. The specific surface area and the surface size of RHA, the w/c proportion, the existence of certain other SCMs, the cement shape, the structural and molecular content of the binder, and the SCM used all regulate the ideal cement substitution of RHA [55,67,68]. The type and dosage of super plasticizer, the desired mechanical characteristics, the aggregate sizes in the concrete, the pore volume of the concrete, the amount of preliminary treatment the RHA receives, and ignition are other factors affecting the optimum cement removal.

Jamil et al. [68] revealed that in each form of cement, the optimum substitute percentage ratio of RHA differs as the percentage of C_3_S (tricalcium silicate) and C_2_S (dicalcium silicate) differs with the type of cement and the quantity of $Ca{\left(OH\right)}_{2}$ provided throughout the hydration reaction. Moreover, the researchers found that the effective modification level of RHA may be increased with an increase in the fraction of external molecules in RH specimens and the presence of non-reactive silica content in the RHA. According to the researcher’s average particle diameter, the specific surface area (SSA), cementitious reactions, high porosity, and cement alternatives in the concrete were the main factors controlling the hydration rate. The form of ash, the grinding cycle, the cement modification level, and the contact between the ash and the cement were also found to affect the intensity of RHAC production [69]. The study used ash (type 2) that had been processed at 650 °C with a 240 min grinding time and a 20% to 40% cement substitute with RHA.

The usage of RHA as a SCM in the production of concrete can result in negative effects, such as decreased flowability, a high-water demand, flow blockages, and increased requirements for superplasticizers. A significant reduction in the concrete’s strength at a high level of RHA, low chloride permeability, and the ASR mechanism in alkaline solution are also stated. All such crucial consequences can be minimized by a thorough study of rice husk ash and the RHAC design phase and the use of suitable RHA content in concrete applications.

### 2.2. Use of IW-II in Polymer Concrete

The industrial waste (IW)-II product silica fume (SF) has also been used for numerous construction purposes and utilized as a cement additive and filler material and for curing purposes [70,71,72,73,74,75,76,77]. Better compressive and flexural performances increase the pozzolanic properties, and multi-range macro void effects are just a few of the benefits of using SF in a mix design [74,76,77,78,79]. The improved macro porosity characteristics of SF can enable its use in different concrete technologies, such as the manufacturing of high-porosity cement-based foams and lightweight concrete (LWC). SF has been proven to be beneficial in elevating the ultimate load-carrying capability and improving the impact resistance and quality [70,71,72,73,80]. The ideal SF dosage is 10% to 14% and can also be used in conjunction with several other additives (e.g., steel fibers, nano silica, and recycled aggregates) [70,72,73]. The poor workability is the major disadvantage of employing silica fume as an additive in concrete [81]. SF was also found to be unsuccessful at reducing creep [82] and resulted in a decrease in compressive strength [83]. Excessive steel corrosion prompted by chloride was also reported in a marine situation and was corrected by using a lower ratio of water to cement [84].

### 2.3. Use of IW-III in Polymer Concrete

The incorporation of the industrial waste (IW)-III product fly ash as a cementitious material in concrete and in different applications has been studied by several researchers [85,86,87,88,89,90,91]. The advantages of using fly ash include an increase in mechanical properties, apparent density, and linear shrinkage, a reduction in pore volume, and enhanced bending strength and toughness [85,86]. The soaking period, soaking temperature, and form of the material used in the fly-ash composite (FAC) should all be carefully chosen to ensure promising applications [88,89]. Optimum design practices must be used depending on the climatic conditions of the FAC material [87,88,92,93]. It is possible to use anthracite or lignite (bituminous coal) as fly ash [94].

The adverse effects of high-volume fly ash concrete (HVFAC) include increased setting times, low strength at initial stages, project delays, cold weather concreting issues, and low resistance to sodium silicate salt carbonation [95]. To prevent the compressive strength from slowly being reduced, Kurad et al. [96] advised that a high percentage of RHA should not be used in concrete production. Additionally, the use of fly ash (high-class C) in silica fume concrete (SFC) can enhance the ASR [97].

### 2.4. Use of IW-IV in Polymer Concrete

The industrial waste (IW)-IV product ground-granulated blast furnace slag (GGBFS) is used in geopolymer concrete (GPC) and alkaline-activated slag (AAS)-based cement production [98,99,100]. An improvement in performance and a high CS are the only advantages of using SF in concrete mixes [101,102]. Chidiac and Panesar [99,103] proposed an optimal ratio of 4:1 (OPC/GGBFS) with a water to binder ratio of 0.3 and a cement to sand ratio of 1:1.5. To resolve bleeding and shrinkage issues, and to ensure a better CS, low alternative proportions and low water to binder (w/b) proportions were recommended [104,105]. GGBFS and fly ash were reported to initiate corrosion and increase critical corrosion. On the flip side, laboratory results and field experience have demonstrated their utility in achieving buildings resilient to severe adverse conditions [106]. Consequently, it has been claimed that if such SCMs are incorporated into concrete production, then there will be no need for additional steel corrosion prevention measures [107]. However, their pairing should be avoided according to experts and adequate measures should also be considered concerning this concrete technology and its particular applications.

### 2.5. Use of IW-V in Polymer Concrete

The industrial waste (IW)-V product waste glass (WG) can be used as a SCM or a filler in a variety of applications, including ultra-lightweight fiber-reinforced concrete (ULFRC) and burned bricks [108,109,110,111,112,113,114]. Additional applications include glass-reinforced doors, concrete building maintenance, and polymer concrete that cures quickly [115,116,117,118]. Enhanced compressive strength, tolerance against freeze–thaw cycles, chloride diffusion, and strong resistance against Na_2_CO_3_ and H_2_SO_4_ are the benefits of using waste glass (WG) [119,120]. The optimal fraction of binder and fine aggregate substitutes was reported to be 5–10% and 7.5–25%, respectively [115,121,122]. A decline in the slump value with high waste glass content and a decline in compressive strength (CS) are the negative effects of using waste glass in concrete [123]. Through valorization, WG can be turned into glass fume by using an appropriate water to cement ratio and waste glass percentage.

## 3. Activation Techniques

An activation process is required to avoid a gradual increase in or low strength at initial stages and to intensify the cementing properties of SCMs in the production of green concrete. Among other benefits, it will gain long-term durability at early or later stages [124]. The activation process has different forms (curing or temperature activation, mechanical activation, SCM-controlled activation, water-controlled activation, and chemical activation), which can easily be found in the literature. Mechanical activation includes crushing a SCM into tiny fine particles to improve their SSA and fineness. The chemical activation process helps to induce the pozzolanic contributions of cementitious composites [125]. Curing/temperature activation implies the usage of a curing form concerning age and temperature to ensure proper concrete development. Air, water, and an alternating combination of both may be used as the curing medium.

To enable the reactivity of the concrete materials, temperature activation tends to refer to temperatures above room temperature. Air and water are common activation channels used during activation driven by temperature. Activation mediated by a SCM requires the utilization of a SCM to intensify pozzolanic effects. Glass particles activated at a high temperature (50 °C) generate pozzolanic actions that also depend upon their composition [126]. The particle size was advised to be smaller than 25 μm. The most effective and feasible activation strategy is chemical activation [127]. Sodium sulfate anhydrite (Na_2_SO_4_), sodium silicate (Na_2_SiO_3_), and acids (HCl and H_2_SO_4_, CaCl_2_, Na_2_SO_4_, NaOH, Na_2_CO_3_, Ca (OH)_2_, K_2_SO_4_, TiO_2_, calcium formate) are a few chemical activators that have been reported in prior studies and used in sustainable and green concrete. Chemical activators may be incorporated into a milling process or coupled with temperature-monitored activation to lessen the overall cost of materials [127]. For example, the combination of grinding and Na_2_SO_4_ addition produced greater strength in comparison with simple activation [128].

Chemical activation has such benefits as a decline in the setting period, strength at early ages, a reduction in material costs, excessive SiO_2_ content, less alkali, an unreactive carbon footprint, a good grindability index, a smaller particle diameter, higher strength, and the development of microstructural features [129,130,131,132]. The chemical activation process is also used in combination with controlled temperature activation. The chemical process increases workability, decreases shrinkage and the weakening of strength at later ages, provides a rapid hydration process, increases the flexural strength properties of SCCs, significantly lowers the pore density, and provides a highly porous structure [133,134]. The author of [135] confirmed that supplementation with nano-based CaCO_3_ by sonication increased the hydration rate, setting period, and compressive strength of SCCs. Intake of colloidal nano-silica achieved a decrease in the initial and final setting times and sometimes increased the CS. Quicklime (CaO) was suggested for high-volume fly-ash-based applications in another study because it provides a significant benefit to both the initial and older-age strength of concrete [136]. The introduction of quicklime into FA-based blended cement improved its early and later strength properties [137]. Lithium (Li) composites were recommended for preventing ASR growth in WGC [138].

SCM-controlled activation was used to increase the bond strength, limit the setting time, attain initial and final-age strength, and lower the bond strength [139,140,141,142,143]. SCMs are widely used and comprise OPC, nano-SiO_2_, and GGBFSS. Bernal et al. [144] proposed activators of silicate derived from SCMs (SF or RHA) as an alternative to outmoded activators in a mixture with aqueous NaOH. The developed binders had mechanical properties that were similar to those of commercially available silicate solutions. Thermal or mechanical activation may be paired in terms of achieving synergistic advantages. The primary advantages include early age strength and the elimination of discrepancies in the mineralogical and chemical compounds of RHA [48,125].

The output of mechanical activation depends upon the activation type used according to a study conducted by Kumar et al. [145]. Their analysis showed that unburned fly ash displayed the peak lime toxicity, followed by fly ash from the vibratory process and fly ash (FA) from an attrition mill. Researchers [146] have proposed a mechanical activation method to improve the SSA and pozzolanic reactions of SCMs before chemical activation. The activator used affects the crystalline structure of the mortars and concretes and the byproducts created as a result [147]. The rank of priority for the alkaline activator was NaOH + WG > NaOH >Na_2_CO_3_, which was derived from the results in terms of CS. In fly-ash binders, they also found that the SiO_2_/Na_2_O ratio and its pH tend to play a key role in cement-based matrix activation and binder strength creation. De Vargas et al. [148] found this to be true and stated that SiO_2_/Na_2_O played an important role in the creation of a geopolymer concrete with FA in terms of improved CS, composition, and microstructure. If the CS concentration rises, the molar concentration rises as well, resulting in longer curing or heat curing times. The inclusion of 5% silica fume to replace the slag in AAS pastes enhanced the compressive strength up to a temperature of 800 °C for SCM-controlled activation [149]. Due to its low solubility and reaction with cement, the use of 5–10% dosages of RHA as a substitute for cement aided in the consumption of free lime, resulting in an enhanced CS-H [150].

In AAS blends for SCM-controlled activation, adding 5% SF as a substitute for slag increased the compressive strength up to a temperature of 800 °C [149]. Because of its direct effect and pozzolanic toxicity, the incorporation of 5–10% RHA as a substitute for cement helped with the intake of free lime, contributing to an improved CS-H [150]. Similarly, the contribution of RHA to SF UHPC caused a low degree of permeability and an improvement in the concrete’s strength of 9.76%, 14.5%, and 10.02% at 3, 28, and 120 days, respectively [151]. The inclusion of nano silica (NS) improved the mechanical efficiency of GPFAC by transforming the amorphous stage of the geopolymer concrete into a crystal-clear stage by geo-polymerization without the use of the temperature activation method.

## 4. Manufacture of Polymer Concrete

The polymer concrete production process varies as it depends entirely on the composite materials to be used as well as the target strength. The authors of [152] proposed four main measures to make ecofriendly and sustainable concrete with satisfactory workability: (1) test the basic properties of the materials used; (2) calculate the w/c ratio concerning the cement % and desired strength; (3) evaluate the size distribution of the mineral composition by a grain size analysis; and (4) perform an experimental evaluation of the fresh concrete’s properties and the physical properties to determine the performance in terms of CS.

The performance of particle packing by a granular inspection of all structural members is one of the evaluation techniques that can be used in green concrete [152,153]. Other techniques include statistical analysis of datasets from microanalysis and C-S-H content estimation [154] and the stepwise design approach [155,156]. Techniques for optimization include particle proportioning of fine aggregates [157], a particle distribution curve [158], optimization based on the response surface methodology (RSM) [159], response surface optimization based on a Box–Behnken design [160], RSM techniques that employ a design tool [161], and a multi-criterion methodology from different aspects such as methodological, cost-effectiveness, and ecofriendly concrete aspects [162]. The removal of air voids is one of the possible benefits of optimizing green concrete, resulting in increased stability and an enhancement of structural properties. Furthermore, Binici et al. [163] suggest sequentially refining one of the SCMs in ternary mixed cement concrete to achieve better compressive strength.

## 5. Properties of Polymer Concrete

### 5.1. Properties of Fresh Concrete

#### 5.1.1. Workability

The slump test is used to determine how compacted concrete behaves in a cone when subjected to gravitational force. This test is also a measure of the strength of the fresh concrete. Bentz et al. [164] suggested that proper and suitable proportioning, testing, and high-range water-reducing admixtures be chosen in order to improve the volume fraction of the aggregate to produce HVFAC. Alaka and Oyedele [165] achieved HVFAC with strong workability using a superabundant superplasticizer (SP) dosage at a low water to binder ratio. According to reports by Yijin et al. [166] and Mukherjee et al. [167], the slump value was significantly increased with a change in the fly ash content. In comparison with Portland cement, FA-blended concrete has a high SSA and low density.

Conversely, Keertana and Gobhiga [168] found that as the RHA percentage increased, the slump values decreased. Similarly, Abalaka et al. [169] found that the slump values increased with the substitution of up to 5% RHA and then decreased. According to a report by Hunchate et al. [170], slump values were increased with up to a 10% replacement of SF with ordinary cement; however, Amarkhail [171] achieved a decrease in slump values with a cement replacement of up to 15% SF. The slump values for fresh GGBFS-based concrete were increased with GGBFS induction. According to research by Tamilarasan et al. [172], maximum slump values are achieved with the incorporation of 55% GGBFS content as a cement replacement in a concrete mixture of grade 20 and 25. A decrease in the slump values of RHA-based fresh concrete was noted because of water absorption. Using SP is a useful way to improve the workability of fresh RHA-based concrete.

The increased water absorption and high specific surface area of RHA material resulted in a reduction in slump [173]. This could have been due to the w/c ratio, the percentage of cement replacement, the hydration process, or the fineness degree [173,174]. It was observed that materials that served as a cement replacement varied the water to binder ratio to achieve a quick gain in strength [169]. Additionally, SF has a relatively low viscosity and yielding stress as compared with RHA. SF particles are spherical and RHA particles are angular. The slump values of a RHA-based mix were found to be lower than those of a SF-based mix. Similarly, it was found that the SF mix showed higher slump values. This was due to the spherical shape of its particles, the removal of absorbed water, and the increase in pore volume [175]. The use of waste glass (WG) material as a substitute for fine aggregate improved the slump values of a WG blend according to Malik et al. [176] and Liang et al. [177]. Abdallah and Fan [178] found a decrease in slump values. This could have been due to variations in the grade of the concrete, the physical properties of the materials, or the percentage of cement replacement incorporated.

#### 5.1.2. Segregation Index

The amount of time it takes to transport, place, and compact concrete is known as the setting time [179]. It was found that the hardening time of GGBFS varies due to the particle diameter, calcium content, and silica/alumina ratio. The initial setting time is 109–141 min and the final setting time is 155–327 min. Researchers [180] have recorded setting times, both initial and final, that range between 4.50–7.45 and 6.30–10.15 h. A SCC based on FA was found to require 3 to 4 h more as compared with control samples. According to research by Brooks et al. [181], no linear relationship exists between the setting time and the percentage of materials used as SCMs. SF findings have been considered to be equivalent to those for OPC and superior to those for FA and GGBSS based on the disparity between the setting times.

Based on the shape and quantity of FA used during concrete construction, researchers [182] found a delay in the setting time that ranged from 4 h 20 min to 5 h 15 min. This delay was due to the presence of sulfate and alkali contents in the cement. Another study [183] found setting times between 145–170 min and 215–235 min. It was found that SF induction increased the initial setting time of the concrete mix, but the final setting time was reduced with the addition of 5% to 10% SF to the concrete.

The strength properties and workability of a RHA-blended concrete mix were improved with the incorporation of up to 30% RHA as a cement replacement according to a study by Ikpong and Okpala [174]. The initial setting time varied from 2 h to 3.5 h and the final setting time was between 4 h and 4.5 h. By using 10% to 40% waste glass content, Lin et al. [184] found an initial setting time of 666–1158 min and a final setting time of 765–1245 min. A decrease in setting time was found with 50% WG content as a replacement for cement at a 0.485 water/binder ratio in a mortar mix [185]. A delay in the setting time and high workability were observed in a mixture of coarse and fine WG. With the use of WG in a concrete mix, we can achieve better results. Its workability, impermeability, and strength properties also improve under high temperatures. Based on research by Bouzoubaa and Lachemi [180], good deformability and stability are shown by FA-based SCCs. With a decrease in water content, an increased flow time has been observed. With increasing FA content, the segregation index was observed to reduce but was observed to elevate with a dosage of SP. To obtain a segregation-resistant FA-SCC, a w/b = 0.45 was suggested. Shen [186] stated that dynamic segregation can be reduced by selecting an aggregate with a small particle size and low density, which will improve the aggregate paste bond.

Fresh properties of SCC produced with ultra-pulverized fly ash (UPFA) have also been examined and should satisfy the following criteria: slump test, 240 to 270 mm; slump flow, 600 to 750 mm; and L-box test, 35 to 80 m/s, as recommended by Xie et al. [187]. When the velocity in the L-box test is >80 m/s, then it difficult to control the segregation and when the velocity is <35 m/s, then the concrete will not satisfy the SCC criteria. The following were suggested for developing SCC-based UPFA: powder content, 500–600 kg/m^3^; sand content, 40%; normal SCM content, 500 kg/m^3^; UPFA content, between 30% and 40%; and a suitable amount of superplasticizer. Generally, proper consideration should be given to ensure that the SCC is capable of keeping its desired fresh properties, such as flowability, permeability, prevention of segregation, reasonable viscosity, and the ability to flow under its weight by using an aggregate of small size.

RHA generated by unregulated burning could be used in new residential building projects according to [188]. Concrete with up to 40% RHA displayed 0.04–8.2% sieve segregation, a 580–670 mm slump flow, a flow time through a V-funnel of 5.9–7 s, and a passing ability of 3.5–5.2, which ensured the satisfactory performance of the SCC’s design. It was verified by Wu et al. [189] that using fly ash in SCC production produces good fluidity (workability). Moreover, the segregation ratio was between 4.4% and 5.6% and the segregation index was 2.9–4.2%. These values are less than 15% and fall into the standard range. Yazıcı [190] observed the minimum value of filling ability at 30% and 40% pozzolanic replacement with SF. The maximum filling ability value was observed at 50% and the same filling ability at 60% cement replacement. Bingöl and Tohumcu [191] found that, in self-compacting concrete (SCC), FA provided a good filling and passing ability as compared with SF. FA-based SCC has been used in many concrete structures, e.g., walls and columns. Similarly, SF-based SCC has been used in lightweight concrete structures. SCC prepared with ternary and quaternary cement has been used in various concrete applications to enhance its fresh properties as per EFNARC specifications [192].

Workability refers to the ability to handle, place, compact, and finish a concrete mix [139]. The addition of GGBFS and fly ash contents to concrete resulted in a decrease in workability, which was attributed to the rapid reactivity of calcium and the geometric features of both materials used. Up to 10% silica fume can be used as a cement additive without losing the workability properties of SFC [193]. Msinjili et al. [194] stated that by using polycarboxylate ethers and lignosulphonate, the workability of fresh concrete could be enhanced. The utilization of bio-additives was proposed by Karthik et al. [195] and showed better fresh properties. Likewise, Ismail and Waliuddin [196] stated that the workability was under the standard limit with the addition of up to 20% RHA. Conversely, Khatri et al. [81] stated that SF slightly reduced the workability of concrete but made a significant contribution towards better mechanical properties.

Hot weather conditions influence the characteristics of fresh cement pastes and concrete [197]. Similarly, with an increase in the curing temperature, IST and FST decrease. The authors of [198] reported that the use of 40% GGBS together with 20% pozzolana achieved successful results, especially in warm conditions. The fresh properties of WGC, such as the passing ability and flowability, increased with increasing WG content [199]. At cement contents of 350 kg/m^3^, 400 kg/m^3^, and 450 kg/m^3^, the flow values were found to be 670–880 mm, 670–740 mm, and 670–780, respectively.

A waste solid within the range 0.37 to 0.41 with a cement content of <400 kg/m^3^ was developed to prevent a rapid initial concrete setting time and a substantial strength reduction [200]. The workability of SCC was increased by using a superplasticizer (polycarboxylate) with 15% GGBFS content [201]. The number of additives should be carefully selected to avoid a loss of strength. The separation of the ingredients of a concrete is termed ‘bleeding’, which is noticeable when there is water on the concrete surface mix [202]. It is affected by various factors, such as the average size of cement particles and the reactivity of the cement [203]. Likewise, several fractions of GGBS and binder contents were introduced to examine the bleeding behavior of fresh concrete.

### 5.2. Properties of Hardened Concrete

#### 5.2.1. Compressive Strength

**Figure 9:**
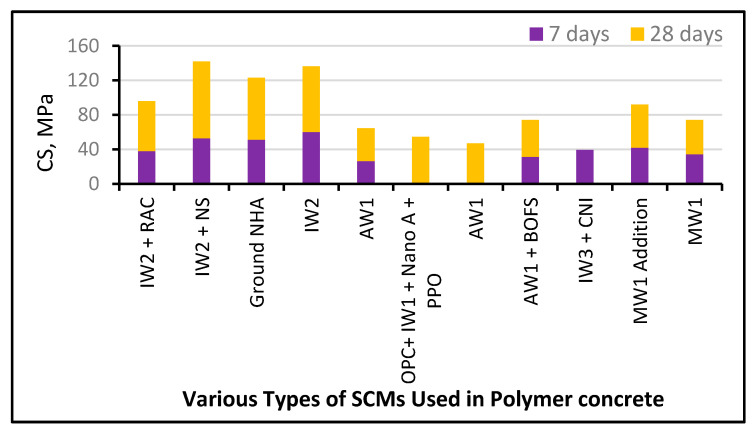
Compressive strength values for polymer concrete mixes for different SCMs.

For a better comparative analysis, the compressive strength (CS) observed in various green concretes by various authors is presented in Figure 9 and the materials used are listed in Table 1. After 90 days of curing, the maximum CSs were achieved (92.1 N/mm^2^, 80 N/mm^2^, and 79 N/mm^2^) by incorporating SF, RHA, and SF with nano silica [72,204]. SF was successfully used to achieve a concrete-like CS of 82.9 N/mm^2^ after curing for 28 days. The inclusion of lime in HVFAC and a binder in geopolymers is used to attain their CS efficiently [92]. Due to their higher Si/Al ratio, geopolymers produced with an alkaline activator were noticed to have a low CS [50,204]. The authors suggested that a suitable combination of NaOH concentration and RHA content depends on production of the compressive strength [50]. Various researchers reported different CSs at different Ca/Si proportions. They reported a CS of 0.2 MPa at a 0.106 Ca/Si ratio [205]. Likewise, they reported a CS of 63.7 MPa at a 0.89 Ca/Si ratio [150]. Chindaprasirt et al. [206] documented a CS < 38 MPa before and after geo-polymerization at Ca/Si ratios of 7.98 and 0.026. It can be further stated that an increased CS is obtained at an intermediate Ca/Si ratio of between 0.85 and 1.0. Scientifically compatible waste products are being used to yield optimal results in blended concrete applications. Different materials with binder properties have been studied to serve as replacement to OPC as eco-friendly economic development of concrete has remained prime target of researchers to follow the circular economy principles. Different materials like RHA, FA, SF, BA, NL, SBR, BOFS, GGBFS and different other materials have been tested for utilization of raw materials as binder [56,70,72,92,204,205,206,207,208,209,210,211,212,213,214,215,216,217,218,219,220,221,222,223,224].

#### 5.2.2. Flexural Strength

**Figure 10:**
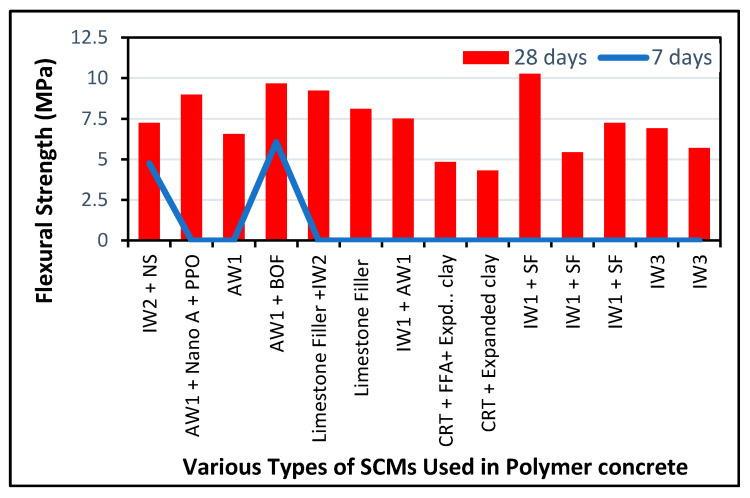
Flexural strength on different curing days for different SCMs utilized in polymer concrete composites.

Many articles on the flexural behavior of green concrete can be found in the literature, as shown in Figure 10. Table 2 lists materials used as SCMs. Mohseni et al. [207] achieved a maximum flexural strength of 10.97 N/mm^2^ with cement + RHA + Nano A + PPO by the quaternary method. This result is supported by another study that used OPC + 20% of FA + 1.5% of steel fibers + a water-reducing admixture. Later on, they used cement + FA + RHA with the ternary method. Walczak et al. [208] observed the minimum flexural strength with waste glass.

Figure 10 shows the results of numerous studies on flexural strength for various types of green concrete, while Table 2 shows the ingredients. Mohseni et al. [207] achieved a maximum flexural property of 10.97 MPa with cement + RHA + Nano A + PPO by the quaternary method. This result is supported by [209], which modified concrete with various material proportions (cement + 20% FA + 1.5% steel fibers + a water-reducing admixture). Afterward, Sathawane et al. [210] blended ordinary concrete with OPC + FA + RHA with the ternary method. Walczak et al. [208] observed the minimum flexural strength with waste glass. The changes in flexural strength could be associated with alterations in the mix, pre-loading conditions, compressive characteristics, SCM, and crushed rock used.

The highest flexural strength was achieved using a steel fiber mortar made with polypropylene fibers, RHA, and nano-alumina. Polypropylene fibers increased the compressive strength of the mortar paste by creating a bridging effect, increasing the fracture energy and thus the flexural qualities of the mortar. Conversely, the capacity of the concrete matrix to load the fibers was improved by nano-Al_2_O_3_ (NA). These synergistic interactions also activated the superior properties of the steel fiber mortar. The prestressed steel-fiber-reinforced concrete in beams attained the second highest flexural strength, which showed a similar pattern. This means that the addition of fibers strengthens the energy absorption efficiency of structural members and increases their overall flexural strength. The flexural properties of concrete can be improved by using various fibers, such as reinforced fibers, polypropylene fibers, and nano-Al_2_O_3_, as reported in several studies [211,212,213]. The lowest flexural strength was found for the case of waste glass mortar and FA. The bridging effect of the fibers and the load transfer issues induced by nano-alumina were absent from this concrete mixture.

#### 5.2.3. Elastic Modulus and Split Tensile Strength

**Figure 11:**
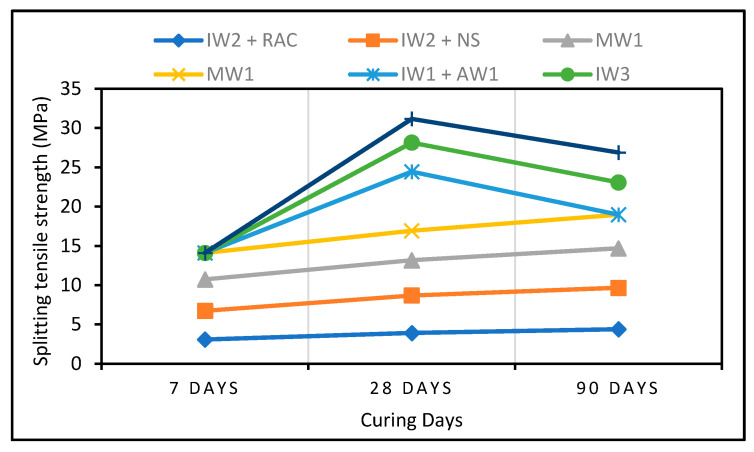
Splitting tensile strength values for polymer concrete mixes for different SCMs.

Figure 11 illustrates the tensile strength of polymer concrete found by several researchers. Jalal et al. [72] obtained a maximum STS of 5.3 MPa using SF and NS, accompanied by 5.07 MPa achieved with waste glass [92]. The low splitting tensile strength (STS) observed in FA-blended cement was correlated with a change in the quality of the ITZ [90]. They suggested curing ages of 50 to 90 days to satisfy the specifications required by the production of lightweight concrete [161]. The elastic modulus of SF-based concrete can be improved further by increasing the SF content [214]. The authors recommended 5% RHA to achieve a higher elastic modulus as shown in Figure 11 [122]. Likewise, they recorded an increased elastic modulus with increasing rice husk contents, found the optimal replacement of RHA cement to be 15%, and showed a maximum CS of 6.70 MPa [215]. Samples produced at different w/b ratios with 10% RHA had a greater overall elastic modulus as compared with those produced with 20% RHA. On the other hand, the authors of [216] reported a decrease in the elastic modulus under a high temperature when GGBFS was used. The higher elastic modulus was achieved at different curing ages and waste-glass-based concrete showed a higher elastic modulus with increasing WG content as compared with natural glass [178].

#### 5.2.4. Shrinkage and Creep

Authors have reported that shrinkage is increased with increasing brick dust content because brick dust can absorb water [225]. It was found that shrinkage was reduced by 33% in the presence of fly ash, which also reduced the concrete’s weight and increased its strength by up to 20% [226]. When compared to type A bricks, shrinkage of 40% in WG bricks was reduced to 50.8%. Because WG has a large percentage of silica particles, it has a lower plasticity index and shrinkage. Likewise, another study showed the same shrinkage values at 30% and 40% replacement with WG. Since the number of finer particles in the brick specimen is increased, the shrinkage may have an equivalent value without a declining trend. The findings of prior experiments revealed that linear shrinkage dropped by up to 25% as the WG content increased [227,228]. Fly ash is used in many applications, such as precast elements, with limited transport costs. It can be used for the development of superior and lighter sustainable and green concrete. To avoid shrinkage, authors have suggested that a suitable aggregate be chosen when making sustainable green concrete [229].

**Figure 12:**
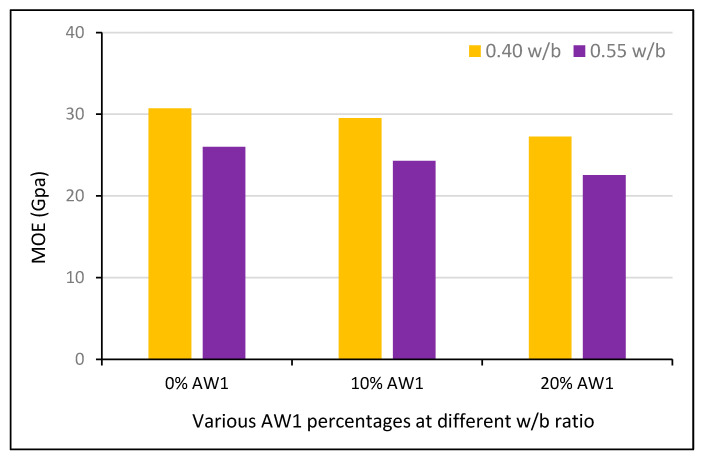
Elastic modulus of RHA-modified concrete at various w/b ratios [233].

**Figure 13:**
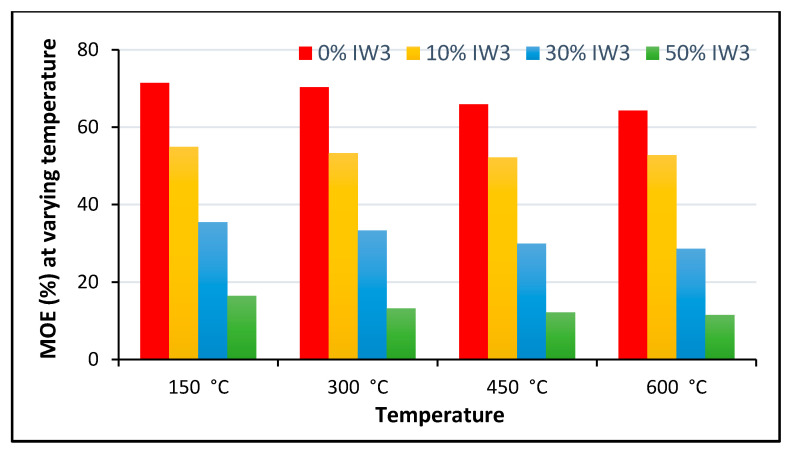
Reduction in elastic modulus at various temperatures and various industrial waste contents [234].

The authors of [230] reported four states of shrinkage: drying, autogenous, plastic, and carbonation shrinkage. It was found that plastic and carbonation shrinkage are caused by improper curing and carbonation reactions, while autogenous and drying shrinkage are influenced by insufficient moisture and drying. Creep is another property that is greatly influenced by the internal relative humidity. It can increase with increasing RHA content. To mitigate the shrinkage and cracking potential of UHPCC, incorporation of 10% SF was recommended [231]. For optimal creep reduction, 15% RHA was suggested. Researchers have found a 55–60% reduction in creep by utilizing up to 55–65% fly ash in HVFA [232]. Elastic modulus of RHA-modified concrete at various w/b ratios shows that lower water to binder ratio shows higher value and can be maintained even after addition of modifier to binders [233] as shown in Figure 12. Furthermore, with the increase in temperature conditions modifiers are not supportive in case of modification to binders and with the increase in extreme temperature condition up to 600 °C MOE reduces [234] as shown in Figure 13.

The need for pre-wetted LWAs in HVFAC was proposed by Barrett et al. [235] to trigger an internal curing process that leads to improved early age strength and a decrease in autogenous shrinkage and tensile stresses. Atis [236] presented research on the utilization of HVFAC, which can be used in flexible pavement and large industrial floors with reduced shrinkage and water use in comparison with cement. Researchers have reported on the utilization of lime water and fly ash powder to enhance the performance of HVFAC, which has low strength. A nonlinear relationship was observed between drying shrinkage and relative humidity [237]. Aggregate proportions and the maximum aggregate size have been reported to be contributing factors that influence shrinkage strain. With increasing aggregate size, a nonlinear relationship develops. As compared with heated specimens, the drying shrinkage strain of environmental control specimens was found to be superior [238].

The use of quaternary cement mixtures (FA, slag, and limestone) was suggested by Serdar et al. [230] to achieve a shrinkage of concrete and a creep deformation of materials comparable to those of cement and to reduce the harmful effects of supplementary cementitious materials in concrete. Moreover, the authors said that the specific creep was greater in OPC as compared with FA-based GPC and was caused by the block polymerization concept [238]. An increase in creep resistance has been observed in FAGPC in comparison with PCC because the performance of fly ash is greatly linked to such a concept. It was noticed that the specific creep was reduced with an increase in CS. Folliard et al. [239] documented this relationship as well. The authors claimed that the creep at early ages appears to be higher in strength at later ages. With an increasing CS, several variables, such as the creep coefficient and the specific creep of FAGPC, were also reduced. Due to the slow strength growth, a high degree of creep deformation was found at the initial curing of HVFAC [96,240]. In HVFAC, the micro-aggregate effect is responsible for the lowest creep deformation, which is due to the residue of FA. The combined effect of SP and HVFA has been reported to reduce creep by as much as 50% [240]. Creep deformation and shrinkage are linked to a loss of water absorption, stress deformations, the refinement of pores, and an increase in small/fine pores as well as the development of the ITZ microstructure [81]. 

**Figure 14:**
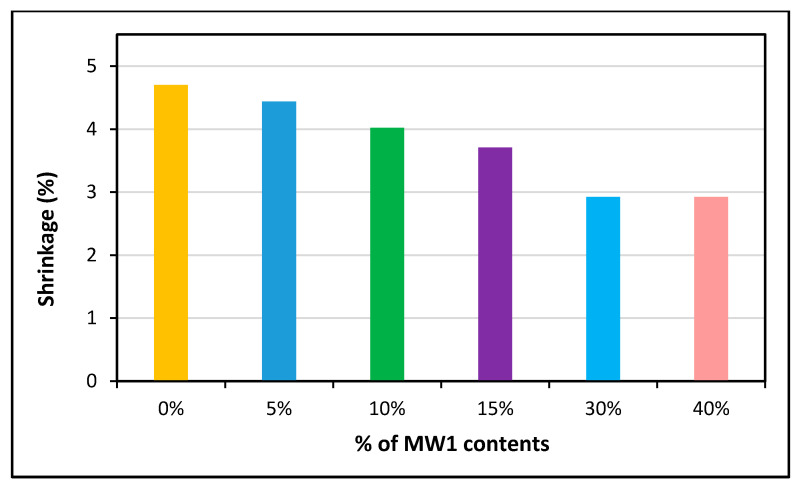
Influence of waste glass content on shrinkage [246].

Based on research, it has been proven that the addition of fly ash causes a decline in creep because of the increase in the modulus of elasticity, which also leads to a reduction in the volume of the paste in the concrete [241]. For cement substitutions with high fly ash content, researchers [242] found increased creep deformation, while another study recommends using 15% FA to achieve desirable properties in terms of shrinkage, elasticity modulus, and CS [243]. Other studies have found that FAC showed similar trends of CS and creep deformation during the hardening process when FA contents between 17% and 33% were used [244]. Moreover, creep deformation is increased with an increase in FA content. Water is also another major factor; however, the addition of SF can be useful to control its movement [231]. The authors confirmed that there is no relationship between creep and shrinkage. Tensile creep outperforms compressive creep by 2 to 3 times, and both are affected by the ambient temperature [245]. In contrast, a researcher reported that, under different applied stresses, tensile creep had an indirect relationship with CS and found that micro cracks due to tensile creep may be improved using the ITZ [220]. At all curing ages, SF reduced the long-term specific creep and drying shrinkage [246] a shown in Figure 14.

### 5.3. Durability Properties of Concrete

#### 5.3.1. Porosity and Water Absorption

**Figure 15:**
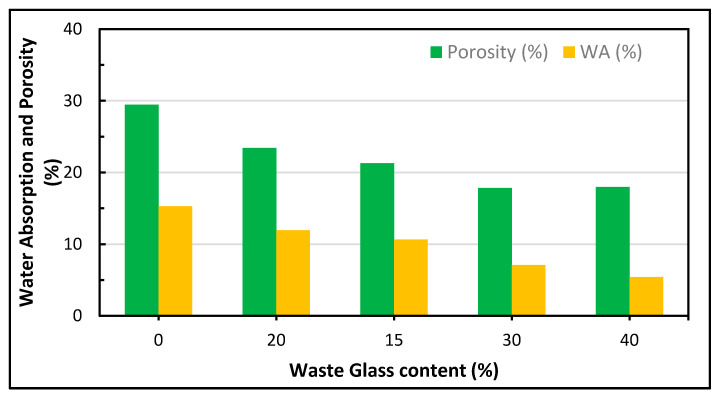
Influence of WG addition on water absorption and porosity [246].

To prevent shrinkage, Yang et al. [220] proposed the optimum percentage of binder substitute to be 60%. Furthermore, the water absorption of cementitious materials, such as RHA and BOF, was investigated in this study and it was noted to be less than that of the fly ash and limestone ternary mixtures produced in another study [90]. The water content decreased as the recycled glass content increased, and the density decreased [247]. A decrease in the shrinkage and porosity of WG was achieved by using cement replacers as shown in Figure 15 [246].

**Figure 16:**
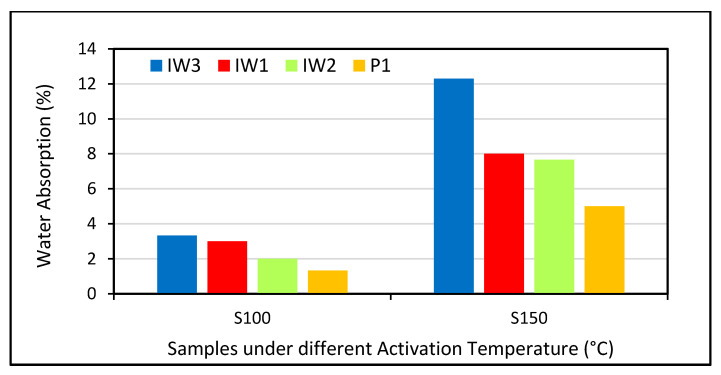
Water absorption (WA) for various materials at different activation temperatures (°C) [248].

**Figure 17:**
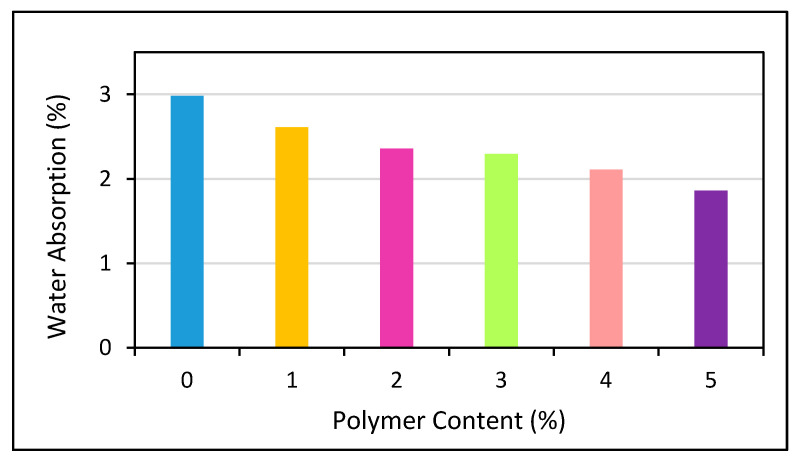
Water absorption of a concrete mix at various polymer to cement ratios [251].

**Figure 18:**
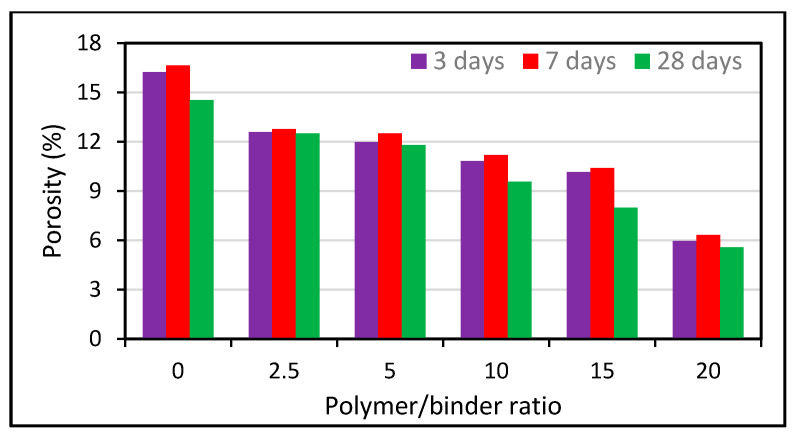
Porosity of a polymer-modified mix at various polymer to cement ratios and curing days [252].

Moreover, it was observed that an improved pozzolanic and pore refinement effect produces a significant reduction in the water absorption and void ratio with an increment in the waste glass powder addition [92]. A study conducted by Binici [248] showed that with an increase in the alkali activation temperature, water absorption decreases, but the decrease varies from one condition to another, as shown in Figure 15 and Figure 16. The effects of varying curing ages and the addition of fly ash at different percentages were also studied and the authors observed a change in the water absorption and porosity values [249]. Increasing the content of cement replacers influences the mechanical properties of polymer concrete due to variations in their porosity and water absorption as shown in Figure 17 and Figure 18. 

In addition, when the polymeric percentage of concrete is increased, impact on water absorption and porosity has also been discussed [250,251,252], the water absorption of the concrete decreases due to pore blocking by polymer particles. Additionally, since polymeric materials are water-impermeable products, the polymer particles that are distributed in the pore spaces will prevent water from infiltrating into the concrete particles. As the polymer content in the mixtures rises, the overall porosity of the materials falls gradually as shown in Figure 18. Increasing the polymer concentration or polymer/cement proportion improves the effectiveness of the polymer filling or wrapping. The utilization of RHA in combination with waste glass, steel fibers, and PPS produces a significant reduction in the void ratio under different w/c ratios. Moreover, a maximum RHA content of up to 10% with a 0.33 w/c ratio was recommended in this study [55]. The use of waste tire rubber and polypropylene fibers causes a loss of water absorption in RHA-based cement composites [250].

**Figure 19:**
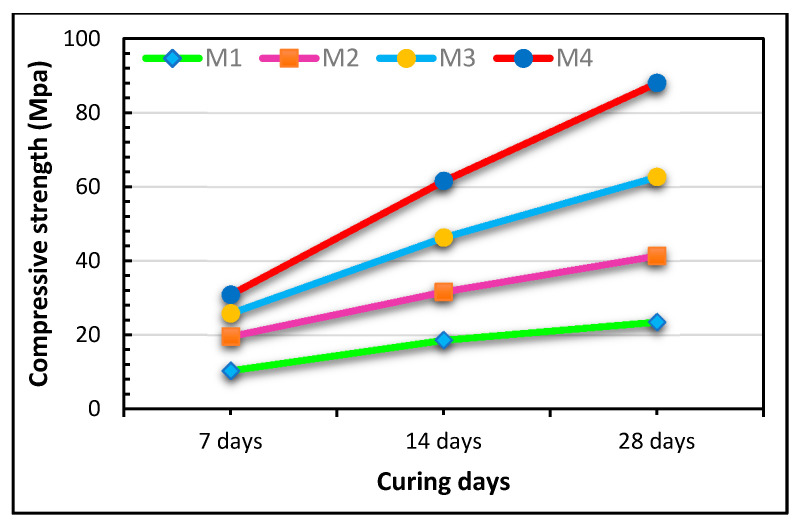
Compressive strength of various IW3 cement replacements on various curing days [253].

Considering the different curing conditions, the compressive strength of the cement mortars made from different combinations of PC, GGBS and PFA at different curing ages. It shows that with the increase in duration of curing time, the CS increase with the increase of GGBS and PFA content for mortar development [253] and are presented in Figure 19.

#### 5.3.2. Alkali Silica Reaction and Chloride Penetration

The replacement of rice husk ash in concrete improves its resistance to chloride attacks [254]. It was concluded that satisfactory performance is achieved with up to 10% RHA content as a cement replacement in comparison with the results of another study as shown in Figure 17 [221]. Likewise, the w/c ratio has a significant effect on chloride ion penetration. It is recommended that a low w/c ratio be used to control chloride attacks; however, a higher w/c ratio triggers a higher degree of chloride ion penetration in concrete [67]. The use of waste glass with a size of <300 nm does not affect the alkali–silica reaction and the results were within the limit ( <10%) described in ASTM C1260 as compared with a concrete mix made with fly ash and silica fume at a 10% replacement level [247]. In addition, the introduction of higher waste glass content as a sand replacement can be used to control ASR [178]. This occurrence was related to a drop in the alkali, possibly due to the overuse of lime in the powder form of waste glass with silica.

**Figure 20:**
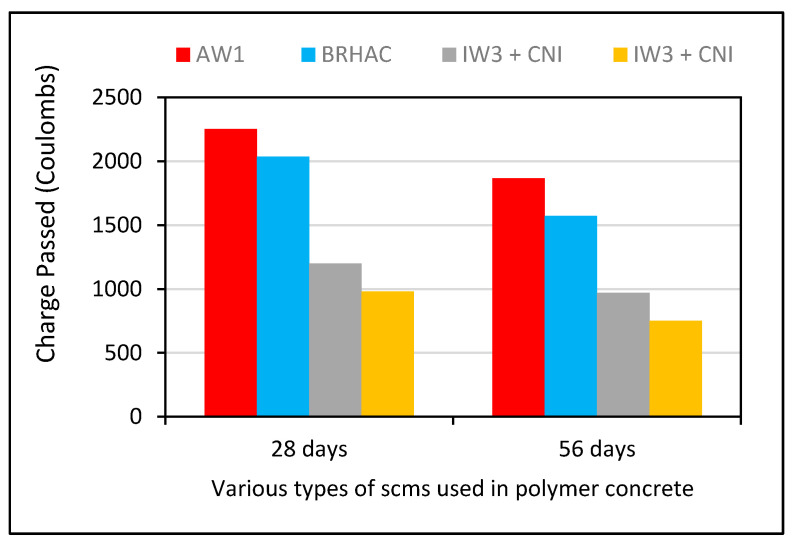
Chloride penetration at various curing ages for different SCMs.

SF shows superior resistance to chloride ion attacks (40% and 14.3% higher than that of RHA). Both were replaced at similar percentages (5% and 10%) with a w/c ratio of 0.6 and tested after 3 days of curing [67]. A decreasing trend has been observed 28 days to 56 days considering the chloride penetration factor as shown in Figure 20. It was observed that SF showed superior mechanical properties in comparison with other additives (fly ash and OPC) at initial stages [255]. Moreover, it showed a similar pattern in comparison to SF when samples were observed after long curing times. Likewise, SF is more useful for reducing permeability (87%) and pore refinement (25%) than CS in HPC. Similarly, when SF was used as a cement replacement at 5%, 10%, and 15%, the chloride penetration observed was 26.7%, 38.5%, and 49.6%, respectively [256].

HPC was prepared with combined mix proportions of 25% RHA and 10% SF [257]. The resistance to chloride ions was reduced by 78.5%, but it was reduced further to 52.36% in the case of a SCC made with a mixture (60% FA + 10% SF) [190]. In HPC, it has been discovered that a 20 mm concrete cover is insufficient to shield steel bars from chloride ion attacks. The use of SF and WG is an effective way to overcome this problem. At 20% WG content, a reduction of 76.85% was achieved [258]. The chloride ion penetration was reduced to 52.47% by utilizing WG in mortar. To achieve the best durability characteristics, a maximum of 10% WG content was suggested [185].

Siddique and Bennacer [202] reported an increase in chloride adsorption affinity with an increase in the content of GGBS [169]; however, the presence of sulfates makes it less efficient. This research employed 60% GGBS as a cement replacement and observed a chloride ion resistance of 81.9% with a 0.55 w/c ratio [259]. The refinement of pores has a significant positive effect on resistance to chloride penetration. By minimizing uncontrolled shrinkage of concrete mixtures, the cracking capability can be reduced [260]. FA-based concrete showed the maximum shrinkage as compared with GGBS and nano silica. Chloride ion resistance is reduced with increasing cement additives, e.g., RHA and SF, for older ages. Similarly, the optimum chloride penetration resistance was achieved in the case of both nano and micro RHA mixtures (2.5% and 12.5%, respectively) [261]. It was observed that a concrete mix made with both types of RHA showed a chloride ion resistance of 71.2% after a 90-day curing process, which was 36.2% more as compared with a sample prepared with 2.5% nano RHA.Based on the chloride ion resistance, one can produce the ranking GGBS > RHA > SF > FA > WG. Research is needed to explain this ideal scenario for resisting chloride penetration by following a mix design, e.g., equivalent cement substitution amounts, water to cement ratio, post-curing sample testing, and other methods for efficient and effective comparative studies. Alkali silica (ASR) degradation is a basic hardness issue in which silica components in aggregate particles respond to broad interactions with alkali pore substances, resulting in crack formation [262]. The performance of any SCM in mitigating ASR varies depending on the composition of the SCM (SiO_2_ and alkaline content), the percentage of SCM, the form of the alkali–aggregate interaction, the amount, and the cement’s fineness [263]. SCMs minimize ASR due to the pozzolanic reaction, which also decreases the permeability of concrete and the use of free alkaline ions by ASR [264].

However, contrary findings on this RHA effect on ASR in concrete have been documented in another study [262]. To control ASR, 12% to 15% RHA was recommended [265]. By the addition of RHA to concrete, an improved ASR was attained [266]. This issue has been solved in another study [267] and it was observed that the diameter of RHA particles is a major factor that prevents and raises the ASR in concrete. Hence, researchers suggested that the cement, equipment, and mixing period must be carefully chosen, as well as the mixing method used. Stable mechanical properties of alkali material (Na_2_O) < 3 kg/m^3^ were observed in another study. Their findings were verified in another study that showed that RHA created by controlled incineration had a stronger ASR inhibitory action similar to that of the remaining RHA induced by uncontrolled burning [268]. A reduction of 51.4% and 82.3% in ASR was observed at 10% and 20% RHA during controlled combustion. Similarly, a reduction of 2.7%, 37.8%, 70.3%, and 94.6% in ASR was recorded in a 10%–40% RHA range in the case of uncontrolled temperature at a 0.47 w/cm ratio in mortar bars.

Moreover, it was observed that SF is more useful for controlling the expansion of ASR in mortar as compared with RHA [266]. A particle size of <5.7 μm was advised in order to exert control over it. The expansion of ASR achieved was 0.01%, 0.02%, 0.02%, 0.06%, and 0.23% at 20% replacement of cement with various waste materials (SF, FA, WG, CRHA, and RRHA), corresponding to percentage declines of 88.9%, 66%, 83.8%, and 37.8% for FA, WG, CRHA, and RRHA, respectively [97,123,268]. Likewise, SF is useful for reducing ASR expansion in concrete as compared with FA [269]. FA and SF replaced 20% of the cement in this study and ASR values of 0.03%, 0.02%, and 0.2 were observed. These findings represent ASR cutbacks of 85 percent and 65 percent, respectively, compared with the control, which gives credibility to the dominance of SF over FA in tackling ASR. ASR values of 0.15% and 0.47% were observed at 30% cement replacement with GGBS, while the control specimen had an ASR reduction of 68.1% [270]. In conclusion, based on above evidence, we can rank the SCMs as follows: SF > FA > CRHA > GGBS > WG > RRHA.

Researchers [271] found that low Ca (calcium) content and increased silica content in a SCM seem to be the most effective at counteracting the alkalinity of cement paste and, finally, the growth of ASR. Researchers have designed preventive methods in line with the environmental conditions, including moisture, alkali interactivity, and temperature, for a sufficient, stimulated, and cheap research methodology. While waste glass was treated as quarry dust as a result of the decrease in the amount of accessible lime, the increase in ASR was observed to decrease in concrete [123]. When WG substitutions of 20%, 15%, and 10% were used as fine aggregates, ASR reductions of 66%, 41.7%, and 16.7% were recorded. Approximately 25% to 100% cement substitutions were examined for ASR expansion and the authors noticed that it relies on the WG percentage and the color of the glass materials [272]. To minimize ASR expansion, they suggested the use of FA and Li_2_CO_3_. The authors also mentioned that the color scheme of the glass had no major effect on the ASR and the thermal stability [273]. They supported the consumption of FA and GGBFS to minimize the expansion of ASR.

#### 5.3.3. Chemical Attacks and Fire Resistance Behavior

The author of [224] studied the impact of a chemical chloride attack on GGBFS concrete mixes with 20 and 40 MPa grades for different curing periods. The CS increased for some concrete in which the acid may chemically react with GGBFS and some other materials. It was highlighted that, with respect to sustainable practices, the substitution of GGBFS with OPC should not exceed 40% and so the acid tends to facilitate pozzolanic effects in GGBFS-modified concrete.

At <300 °C, SF had a significant impact on excess CS. In SFC using 10% SF as a cement substitute, the strength retention was 84.1%, 85.2%, 68.8%, and 26.8% at temperatures of 100, 200, 300, and 400 °C. At 6 percent of cement replacement, the strength quality was higher than the average values of 84.1 percent, 85.2 percent, 68.8 percent, and 26.8 percent [274]. The loss of strength was due to the rupture of the ITZ bond within the aggregate and paste and even the chemical reaction of wetting materials. A strength increase ranging between 1.3 and 3.7 percent was found in all 200 °C concretes. For SF, the author of [275] stated that the strength recovery was 94.5 percent, 60.9 percent, and 47.3 percent, and in the case of RHA at temperatures ranging between 200 and 600 °C the strength retention was 103.6 percent, 46.4 percent, and 48.2 percent. The results showed that SF had better strength durability than RHA. At 800 °C, only the RHA-based concrete had satisfactory strength.

Rashad et al. [276] observed a compressive strength of 45.92 N/mm^2^ at a replacement percentage of 70% with OPC at a 400 °C temperature in the development of HVFAC. It was lower in comparison with the 67 N/mm^2^ and 52 N/mm^2^ for SF content in stimulated alkali paste as observed by the author under similar temperature conditions [275]. In addtion, an overall increase in values of CS across all mixtures with the temperature of 400 °C was observed and was due to matrix densification. An increasing loss of strength was observed in the temperature range from 400 to 1000 °C. Water loss, rising porosity, and permeability were linked to it. Furthermore, compared with neat concrete, HVFAC exhibited improved fire efficiency, while GGBS addition clearly showed negative effects on CS at elevated temperatures. At temperatures between 800 and 1000 °C, FA-GP showed low thermal stability due to the increased average particle size and the substitution of Na-feldspars with a crystal structure [133]. At temperatures ranging between 200 and 1200 °C, a Class F ash-based GP produced using a Na ion channel showed six compressive strengths (12 MPa, 14 MPa, 30 MPa, 33 MPa, 37 MPa, and 38 MPa, respectively). In contrast, FA-GP produced with potassium silicate showed CS degradation above 1000 °C, whereas the amorphous structure persisted. This indicated that, due to dramatic declines in CS and large water losses at both 800 °C and 1200 °C, FA-based GP substances should not be used for a refractory industrial purpose.

At an elevated temperature (800 °C), HSC produced with SF at 15.4 percent and FA with a cement replacement of 38.5 percent showed a significant reduction in CS of 74.4 percent from 97.3 N/mm^2^ to 24.9 N/mm^2^. At the same temperature, control samples showed a 54.7 percent reduction in CS. The disparity in microstructure in both the HSC and control specimens was linked to the degradation of HSC, which incorporates 9 percent SF by weight of cement. A negligible reduction in CS at 100 and 400 °C and a serious decline at 400 °C (approximately between 55 and 80 percent) was reported. The authors of [277] confirmed a deterioration in strength in the temperature range between 100 and 200 °C in HSC by incorporation of a SF dosage of 7.53 percent as a cementitious material at a 0.32 w/c proportion, which was attributed to the polarization of physical properties. The author of [278] claimed that a FA-based GP paste showed a 6 percent higher CS of 62.8 N/mm^2^ and about an 11 percent decrease in volume under a high temperature of 800 °C in comparison with untreated control specimens. The increase in CS was due to its low water absorption, the availability of a significant amount of void space, and possibly the solid content ratio.

For fire safety and performance improvement in GPC, the FA/activator ratio was found to be the most significant factor. The desired ratio of Na_2_SiO_3_ to KOH was 2.5 and in the case of the FA/activator ratio 2.5 was recommended. Both the polymerization reaction and sintering are responsible for an improvement in the strength of GPs at high temperatures. Another research study [279] confirmed that the size of aggregates and the expansion amount are significant factors that influence GPC’s quality at elevated temperatures. Aggregates of < 10 mm in size facilitate cracking and spalling in the concrete matrix, whereas aggregates of > 10 mm in size were found to be safe. The author of [280] recorded a strength shortfall of about 15% in a fine glass powder mortar at temperatures of <500 and 56% at 500 and 800 °C. A drop in the Ca(OH)_2_ in the GP-blended cement paste, melting of the glass fibers, and greater incoherence in the mortar and sand were all linked to a loss of strength.

According to [281], incinerated FA characterized by GGBS performed more effectively at higher temperatures than SF in the preparation of concrete and can be used in significant fire hazard situations. The optimal FA and GGBS substitution rates in HSC and NSC are 30% and 40%, respectively, to attain the desired strength and sustainability [281]. Furthermore, due to explosive spalling, SFC > 5% replacement of cement should be avoided. FA > GGBS > SFBS > SFBS was the sequence of choice for CS production at high temperatures based on the performance. The average intensity reductions in HSC when FA or SF was added, including GGBS-blended NSC, were 44 percent and 60 percent, respectively.

When GGBS is used as a sand substitute in an alkali-activated slag (AAS) mortar, researchers [282] demonstrated higher retained toughness at an elevated temperature. Overall, the compressive strength reductions at a temperature of 800 °C were 33.5%, 51.9%, 69.5% for sand substitution levels of 90%, 25%, 50%, 75%, and 100%, respectively. During the higher-temperature trials, the AAS mortar did not produce a micro-crack. Tanyildizi and Coskun [283] examined LWCs containing FA content ranging from 0 to 30% as cement substitutes under varying temperatures (200, 400, and 800 °C). CS values ranging between 38 and 48 MPa, 35 and 38 MPa, and 14 and 23 MPa were documented under elevated temperatures ranging from 200 to 800 °C, respectively. Similarly, the percentage of strength loss obtained varied from 91.1 to 99%, 80.2 to 93.0%, and 36.1 to 43.6%. The loss of CS was influenced by the water being retained during hydration in hot weather. The splitting tensile strengths obtained varied significantly, from 87.8 to 91.9%, from 81.9 to 85.6%, and from 23.6 to 43.2%, at temperatures ranging from 200 °C to 800 °C. The heating severity and FA concentrations are among the most significant factors on which the STS and CS of FAC depend, according to an ANOVA report with a weighted average of 93.4%, 89.4 %, and 4.8%, respectively. The maximum allowable FA amount to produce the desired CS and STS was 30%.

Concrete made of fine glass waste as a sand replacement was given the highest CS in comparison with coarse WGC and WGC containing a mixture of both fine and coarse particles [284]. The best WG material for achieving the optimum CS in both normal and extreme environments for the three mixture types was the 10% aggregate removal material. The three concrete CSs were attributed close to 700 °C due to their vicinity to the operating temperatures, which range between 700 and 800 °C of WG content. Although the durability of pulverized FAC began to deteriorate at about 250 °C, it showed a significant improvement between 450 and 650 °C [285]. The CS loss was caused by an increase in the ITZ width, an increase in the overall porosity of the structure, and densification of the concrete matrix.

In binary cementitious products, RHA has a higher resistance to a sulfate threat than FA. Despite this, the RHA mortar’s strength improved by 7% after 90 days of immersion in a 5% solution of sodium sulfate and a 20% cement alternative, which compares favorably to 0% for FA [286] In comparison with RHA, which registered a 24.6 percent strength decrease after 90 days at 40 percent cement replacement, fly ash registered a strength increase of 8.8%. The optimal RHA and FA cement replacements to ensure CS stability and an improvement in CS were 20% and 40%, respectively. Researchers [287] suggested that, in order to obtain improvements in the concrete’s strength and enhancements in resistance to HCl and H_2_SO_4_, 20% of the cement be replaced by RHA. The acid attack resilience was directly related to the ratio of (SiO_2_ + Al_2_O_3_ + Fe_2_O_3_)/CaO. RHA’s increased resistance was also attributed to its dense microstructure, its structural and pozzolanic effects, and the existence of Al_2_O_3_. A 25% RHA cement substitution with 0.1H_2_SO_4_ resulted in a strength increase [288].

The immunity of FA and SF to several chemicals (sulphuric acid, nitric acid, acetic acid, phosphorous acid, sodium sulfate, and magnesium sulfate) has been investigated [289]. The authors said that SF was more resistant to cement removal, with a 15% increase in resistance. The SF content showed a 16.6% and 17.8% lower strength loss compared with 23.5% and 38.9% for FA at 15% and 22.5% cement substitution levels, respectively. Chemical immunity is affected by the particle size of FA. CSs were improved from 41.5 N/mm^2^, 53.5 N/mm^2^, 56 N/mm^2^, and 61.5 N/mm^2^ to a better Blaine fineness of 3000 cm^3^/g, 3900 cm^3^/g, 4800 cm^3^/g, and 9300 cm^3^/g, respectively [290]. The optimal degree of substitution for chemical acid exposure varies depending on the type of acid or alkaline substance applied. It was noticed that FA’s chemical acid resistance was more efficient compared with SF at higher substitution levels. The mitigation of sulfate ion caching within concrete has been aligned to sulfate resistance, resulting in a small pattern of gypsum (CaSO_4_) and ferric oxide inside the concrete structure [291]. Chemical resistance rises as the cement content rises and the w/c ratio falls, and cement with 7 percent tricalcium aluminate (C_3_A) content has been used [292].

The chemical susceptibility of GGBS in the presence of lime is determined by its high toxicity, the amount of Ca in the cement matrix, and its dispersion within that sample [293]. GGBS outperformed FA in terms of leaching and sulfate attack resistance [294]. The literature indicates that cement hydration of C_3_S and C_2_S is attributed to the creation of portlandite, which promotes the penetration of sulfate when extracted and produces spacious ingredients (gypsum and ettringite). Similarly, GGBS outperformed FA in terms of resistance to a MgSO_4_ attack due to its higher CS2 content [295]. In concrete, approximately 50% GGBS is being used to achieve strong sulfate resistance properties and mitigate carbonation and permanent deformation [296]. Moreover, concrete containing up to 70% GGBS experienced promising resistance against a Thomasite-type sulfate attack, and its susceptibility was enhanced by adding a suitable amount of CaCO_3_ or CaSO_4_ [297]. Compared with fly ash, GGBS demonstrated high resistance to a sulfate attack and the ideal cement substitution reached 40% for GGBS [298]. Despite its superior resistance, the authors of [299] claimed that GGBS could not be used in drainage structures due to its inability to sustain stress from sulphuric acid strikes. By preserving the weight equilibrium during a sulfate attack, waste glass enhanced WGC’s durability. Furthermore, over 6.7 years of field testing, the performance quality of slabs and walls finished with WG has improved significantly [300]. Glass fume produced by WG fragments is more resilient against sulfate attacks [301].

According to Ganjian and Pouya [302], when exposed to a tidal setting, OPC performed better than SFC, while a combination of SF and GGBS performed badly. Researchers [303] confirmed that sulfate attack resistance was enhanced using mortar produced with quaternary blends, such as GGBS, when as compared with normal concrete. According to Aziz et al. [304], GGBS could increase the performance of sulfate-resistant cement (SRC) by up to 30%, which could be used to make strong and efficient concrete. The change in net pore volume, overall chloride content, overall sulfate content, and free lime content resulted in higher sulfate and chloride ion permittivity, which contributed to the overall performance of the modified concrete.

### 5.4. Circular Economy Model for Polymer Concrete Development

**Figure 21:**
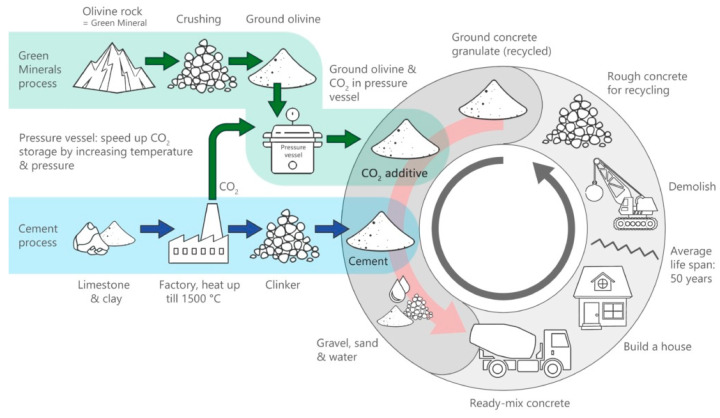
Green Mineral and Regeneration Design—Recycling for Ecofriendly Concrete Development [305].

Materials produced by following an environmentally friendly ideology, such as reducing CO_2_ emissions and using substitutes in cement production, can help in polymer-modified concrete. Two institutes of international repute (Heidelberg Cement and RWTH) have developed an ecofriendly concrete concept using olives and basalt. Using carbonized minerals for the development of construction materials will be helpful. Two institutes, Heidelberg Cement and Aachen University-RWTH, are supported by the Potsdam Institute for Advanced Sustainability Studies (IASS) and the Dutch start-up Green Minerals for this research [305]. Figure 21 presents an idea for concrete development using waste materials as a circular economy concept. Different types of polymer materials including different raw products consisting of binding properties can help with concrete production following the same concept.

## 6. Challenges and Future Directions in Polymer Concrete Composites

A major challenge in the development of polymer concrete composites is ensuring compliance with regulatory standards. Other challenges include minimum clinker concentrations, the chemical characteristics of cement, the insufficiency of the data available in the literature over the last 20 years, and the diversity of polymer concrete applications. Therefore, more research is required for a better understanding of polymer concrete’s behavior [306]. As a result, construction compliance codes must be revised to make them more ecofriendly and promote the use of polymer concrete.

Regulations and inexpensive technology for the efficient development and manufacturing of polymer concrete and relevant information are needed to support and understand the introduction of new standards and regulations [307]. To raise awareness about the potential benefits of polymer concrete, proper technical training and skills should be provided to environmental experts. This will promote the use of polymer concrete composites in the construction sector. Moreover, the challenges experienced by construction or consulting firms should be tackled in this way.

Similarly, innovative and economical activators are needed to promote the long-term advancement and production of PCC. Characterization methods that are both cheap and economical are also desired, particularly in developing economies where funding for research and development is limited. Construction firms, academic institutions, and research centers should be paid to pioneer the manufacture and use of polymer concrete composites in their construction projects. Moreover, new research hubs should also be established to support the continued advancement of polymer concrete materials and construction methods. Domestic production techniques should be supported in order to generate low-cost green composite materials and decrease dependency on costly technology. One of the most productive, economical, novel, and sustainable strategies to boost the effectiveness of concrete buildings is to use waste materials as well as alternative composites, e.g., SCMs and coarse aggregates, in polymer concrete production.

## 7. Conclusions

Polymer concrete composites are made from different types of industrial, municipal, and agricultural wastes. Polymer concrete composites are identical to normal concretes; however, they are made entirely of polymer matrices rather than cement and contain two co-binders, namely mineral cement and a high proportion of polymer. Owing to variations in the development goals, capabilities, and ability levels of the local construction industry, the strategies adopted in each country to promote the use of polymer concrete in buildings will be different. Polymer concrete composites, which incorporate waste products and modern, substitute materials such as SCMs and aggregates, constitute one of the most effective, inexpensive, advanced, and ecofriendly ways to improve the quality of concrete buildings. Polymer concrete composites can be used in large-scale construction projects all over the world. Proper measures and cooperation between building organizations are actively needed to promote the concept of sustainable concrete in construction. Besides this, more demonstration projects, as well as further research and development, are needed to develop environmentally friendly material binders to minimize the need for OPC. For the building industry, polymer concrete is strongly advised due to its natural, technological, and economic advantages.

Moreover, future research should focus on the following. Guidelines for the advancement of resources for polymers that endorse industrial waste could be developed for use across the world. Based on the sources of raw material and the mineralogical nature of industrial materials, a precise model framework for polymer concrete could be established. Due to the disproportionate emission of carbon during the cement manufacturing process, the contribution to global warming of cement factories still alarms environmentalists. As a result, supplemental industrial or agricultural wastes can be used as a raw material to minimize energy consumption. Under the circular economy model, industrially focused polymer design parameters could be defined to establish region-specific and raw resource materials.

## Figures and Tables

**Table 1:** Materials (Binders) utilized in different polymer concretes.

Researcher	Description of Work
Mohseni et al. [56]	RHA and PPO used as a replacement for OPC
Çakır and Sofyanlı [70]	RAC + SF used as a replacement for OPC
Jalal et al. [72]	SF + NS used as a replacement for OPC
Aliabdo et al. [92]	Glass powder used as a replacement for OPC
Xu et al. [204]	RHA used as a replacement for OPC
Liu, B., et al. [217]	FA used as a replacement for OPC
Andayani, S.W., et al. [218]	Natural Latex-KOLAM used as a replacement for OPC
Borhan, and Al Karawi [219]	SBR used as a replacement for OPC
Yang et al. [220]	BOFS + RHA used as a replacement for OPC
Boga et al. [221]	CNI + GGBFS used as a replacement for OPC

**Table 2:** Materials utilized in different polymer-type concretes.

Researcher	Description of Work
Mohseni et al. [56]	Cement+ PPF + RHA used in composite concrete development
Jalal et al. [72]	SF + NS used in composite concrete development
Walczak et al. [208]	CRT + FFA used in composite concrete development
Patil and Sangle [209]	Steel fibers + FA used in composite concrete development
Sathawane et al. [210]	RHA + FA used in composite concrete development
Benaicha et al. [214]	SF + LF used in composite concrete development
Borhan and Al Karawi [219]	SBR used in composite concrete development
Yang et al. [220]	RHA + BOF used in composite concrete development
Esmaeili, J., et al. [222]	Steel fibers used for polymer-modified concrete
Ardalan et al. [223]	Polymer + recycled aggregates used in SSC development
Karri et al. [224]	GGBFS used in composite concrete development

## Data Availability

Data will be made available on reasonable request.

## Nomenclature

AW1	Rice husk ash (RHA)	CS	Compressive strength
AW2	Corncob ash	SF	Steel Fiber
AW3	Sawdust ash	CIP	Chloride ion penetration
IW1	Fly ash (FA)	WA	Water absorption
IW2	Silica fume (SF)	MOE	Modulus of Elasticity
IW3	Granulated Blast furnace slag (GGBFS)	S_eff_	Effective surface area
MW1	Waste glass (WG)	BRAC	Bacterial rice husk ash concrete
MW2	Plastics	MW3	Paper waste
